# Low-Grade Appendiceal Mucinous Neoplasm: A Case Series

**DOI:** 10.7759/cureus.28755

**Published:** 2022-09-03

**Authors:** Andy S Wang, Hussam N Ismael, Jignesh Parikh, Victor L Modesto

**Affiliations:** 1 Surgery, University of Central Florida College of Medicine, Orlando, USA; 2 Surgery, Orlando Veterans Affairs Medical Center, Orlando, USA; 3 Pathology, Orlando Veterans Affairs Medical Center, Orlando, USA

**Keywords:** intraperitoneal chemotherapy, cytoreductive surgery, hemicolectomy, appendectomy, extra-appendiceal mucin, pseudomyxoma peritonei, appendiceal mucinous neoplasm

## Abstract

Low-grade appendiceal mucinous neoplasm (LAMN) is a lesion of the appendix with potentially fatal consequences if untreated. Though LAMN can be asymptomatic and stable, it can rupture and seed mucin and neoplastic epithelium into the peritoneum, leading to pseudomyxoma peritonei (PMP), a serious complication characterized by intraperitoneal accumulation of mucinous tumors and ascites with a high morbidity and mortality rate. Therefore, timely identification and treatment of LAMN are crucial for reducing PMP risk and improving prognosis and outcome. This case series sought to examine five LAMN cases and delineate the strategies for managing LAMN and progression to rupture and PMP.

## Introduction

Low-grade appendiceal mucinous neoplasm (LAMN) is a tumor of the appendix with a risk of serious complications [1-3]. It is an uncommon lesion that can be found during evaluation for unrelated complaints and can present with abdominal pain, vomiting, distention, palpable mass, intestinal obstruction, weight loss, and intussusception [1-3]. They can also uncommonly present with urological findings, including hematuria, ureteral obstruction, hydronephrosis, and urinary tract infection [1, 4]. While LAMN can be stable, they carry a risk of rupturing and seeding mucin and neoplastic cells into the peritoneum and leading to pseudomyxoma peritonei (PMP), an intraperitoneal dissemination of mucinous ascites and tumors that is associated with poor outcome and low survival rate [1-3]. Thus, timely management is essential to prevent LAMN dissemination and progression to PMP and improve overall outcomes and maximize the likelihood of survival and recovery. Given the serious complications of LAMN, this case series sought to evaluate five cases of LAMN to elucidate strategies to manage LAMN and progression to PMP.

## Case presentation

Case 1

A 45-year-old female presented with three days of constant dull left upper quadrant (LUQ) abdominal pain associated with nausea, vomiting, and bloating. She experienced a similar episode one month ago with intermittent LUQ pain, nausea, and vomiting for five days. She did not have fever, dysphagia, constipation, or urinary symptoms. She had LUQ and epigastric tenderness on exam, but no right lower quadrant (RLQ) tenderness, McBurney’s point tenderness, or peritonitis signs. Labs showed a normal leukocyte count. Computed tomography (CT) scan of the abdomen and pelvis with intravenous (IV) contrast revealed a dilated fluid-filled appendix measuring 1.5 cm in diameter with adjacent fat stranding. The patient was offered surgery as it is the standard of care. However, she declined surgery and elected for non-operative management with antibiotics. The patient's wishes were followed under the condition that follow-up imaging would be obtained to evaluate if the findings had improved, and if not, then surgery would be strongly recommended. She was initially managed non-operatively with antibiotics and interval imaging. Repeat CT scan with IV and oral contrast two months later re-demonstrated an unchanged 1.5-cm dilated appendix. A laparoscopic appendectomy was performed for diagnosis and treatment. Pathological examination demonstrated a 1.4-cm well-differentiated LAMN with acellular mucin invading the subserosa or mesoappendix, but not extending to the serosal surface, staged as pT3. Because the histology did not show spread into the peritoneum, appendectomy was considered sufficient for treatment.

Case 2 

A 79-year-old male presented with three weeks of constipation and a 24-pound weight loss over the past year. He denied any family history of colorectal cancer. He was found to have a RLQ mass on exam. Labs demonstrated elevated carcinoembryonic antigen (CEA) levels. CT abdomen and pelvis with IV contrast demonstrated a 7 cm x 13 cm x 8 cm cystic lesion lying adjacent to the cecum consistent with a fluid-filled appendix. Surgery was recommended to the patient as the standard of care, but the patient refused and requested for non-operative management. The patient was managed non-operatively under the condition that surgery would be strongly advised if the findings did not normalize on follow-up imaging. A repeat CT abdomen and pelvis with IV contrast two months later demonstrated a 7 cm x 12.5 cm x 8 cm cystic lesion with peripheral calcification in the right abdomen and a thickened appendix measuring up to 1 cm without peri-appendiceal stranding. Exploratory laparotomy and appendectomy were performed, during which a large cystic appendiceal tumor was removed. Pathological examination of the appendiceal mass revealed an LAMN with negative margins and pTis (in situ) staging. Given the negative margins with no findings of peritoneal dissemination, appendectomy was deemed acceptable for management. Additionally, because LAMN could present with elevated CEA as a tumor marker and was associated with concurrent gastrointestinal (GI) malignancy, a colonoscopy of the cecum was performed, which was negative for signs of colorectal cancer.

Case 3

**Figure 1:**
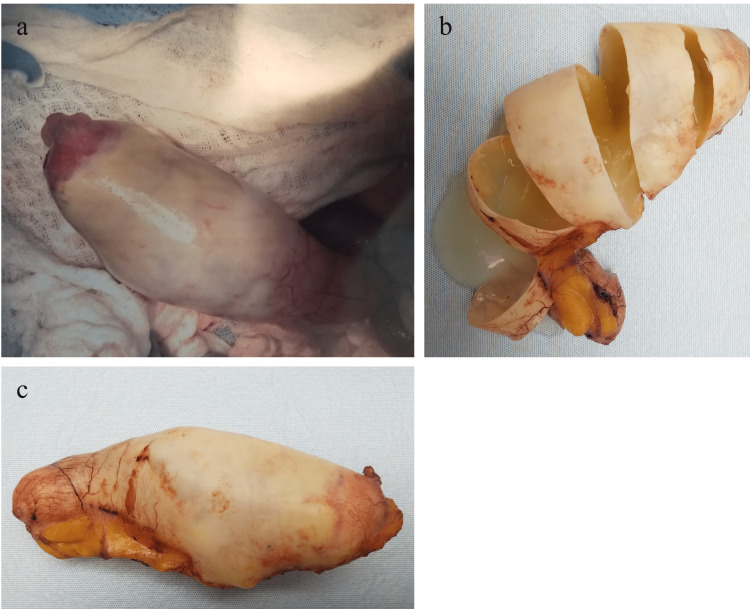
Gross appendiceal specimen of Case 3 (1a-c). Appendix is grossly distended, with cut surfaces showing mucin-filled lumen. Specimens with LAMN, as in this case, can grossly present as dilated mucinous appendices. Pathology of this specimen showed LAMN. LAMN, low-grade appendiceal mucinous neoplasm

A 59-year-old male presented with microscopic hematuria. Renal ultrasound showed a large tubular lesion in the pelvis. CT abdomen and pelvis with IV and oral contrast demonstrated a 14.5 cm x 6.3 cm x 5.6 cm peripherally calcified tubular fluid attenuation lesion in the right hemipelvis. Though surgery was recommended for management, the patient declined surgery and wished to be managed non-operatively. The patient's request was followed, and the patient was counseled that if the findings did not normalize on follow-up imaging after non-operative management, then surgery would be highly recommended. A follow-up CT scan with IV contrast five months later re-demonstrated the RLQ tubular cystic mass measuring 14.4 cm x 5.3 cm in the coronal plane with heterogeneous mural calcifications lying adjacent to the appendiceal tip. An open appendectomy was performed and the cystic appendiceal mass was removed (Figure 1). Because pathological examination demonstrated an LAMN confined to the appendix with an intact serosa and proximal and mesenteric bowel margins negative for neoplasms, staged as pTis, appendectomy alone was considered suitable for treatment.

Case 4

A 68-year-old male presented with microscopic hematuria. CT scan of his abdomen and pelvis with and without IV contrast revealed a 1.6-cm dilated appendix with fluid attenuation material and no surrounding inflammation or wall thickening. A laparoscopic appendectomy was performed. Given pathological examination demonstrated LAMN with pTis staging, appendectomy was considered sufficient for management.

Case 5

A 61-year-old female presented with RLQ and flank pain, anorexia, and a 40-pound weight loss over the past four months. CT scan of her abdomen and pelvis with IV contrast demonstrated an enlarging circumscribed 5.5 cm x 4.2 cm x 4 cm cystic mass in the distal appendix without inflammatory changes. The mass was surgically removed. Pathological examination revealed an LAMN confined to the appendix with uninvolved margins, staged as pTis. 

All patients recovered well and were discharged home with no complications. Their symptoms had largely resolved with no further issues noted at their follow-up appointment one year later. Repeat CT imaging showed no evidence of recurrence so far.

## Discussion

**Figure 2:**
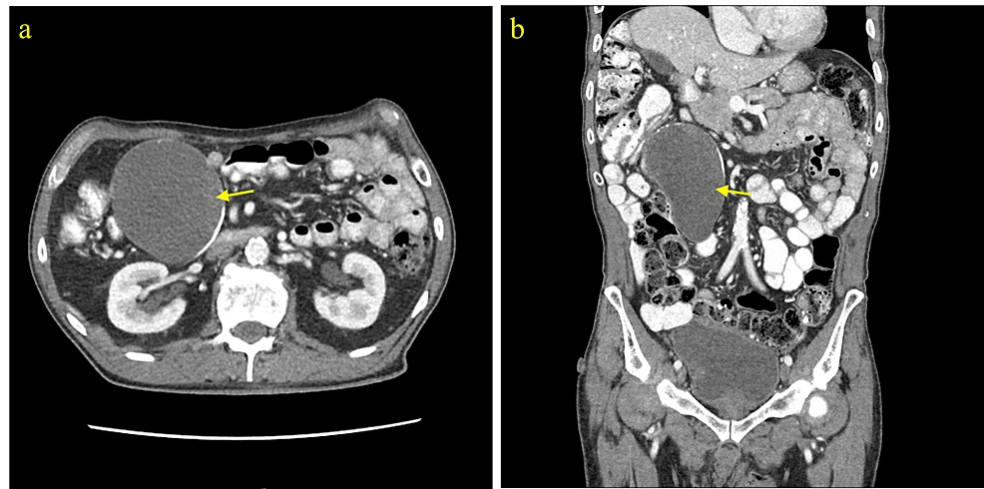
CT abdomen and pelvis with contrast (Case 2). a) Axial view and b) coronal view showing a cystic lesion with peripheral calcification in the right abdomen. Subsequent exploratory laparotomy and appendectomy revealed a large cystic appendiceal tumor. Pathology of the lesion was consistent with LAMN. LAMN, low-grade appendiceal mucinous neoplasm

**Figure 3:**
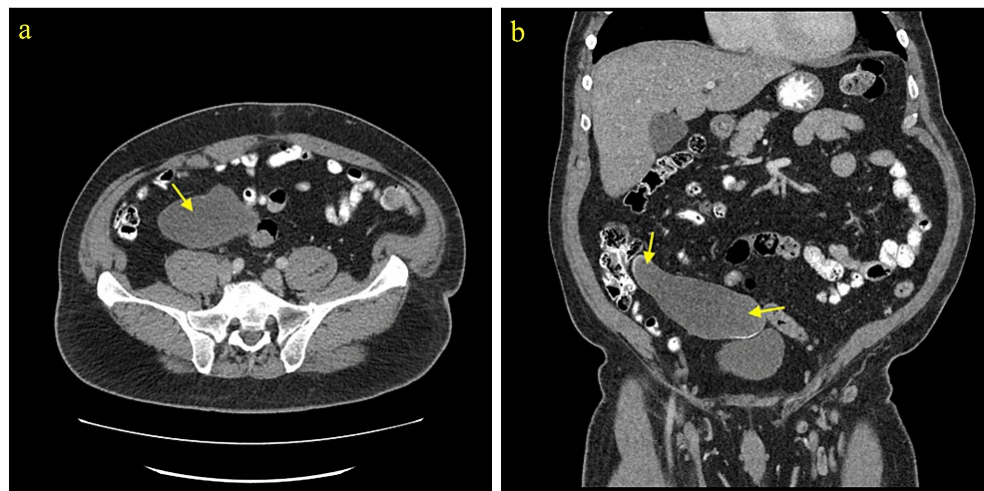
CT abdomen and pelvis with contrast (Case 3). a) Axial view showing a cystic lesion in the right abdomen. b) Coronal view showing a RLQ tubular cystic mass with heterogeneous mural calcifications. Pathology of the mass showed LAMN. RLQ, right lower quadrant; LAMN, low-grade appendiceal mucinous neoplasm

**Figure 4:**
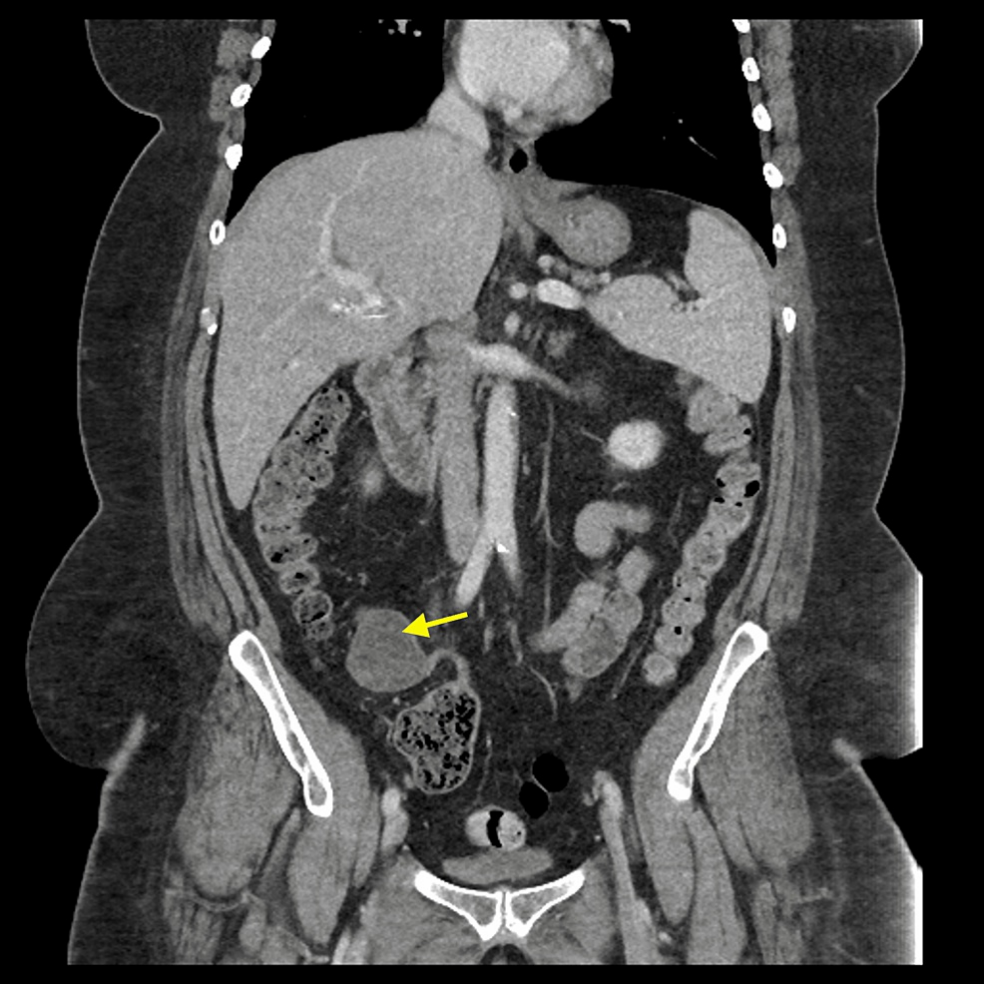
CT abdomen and pelvis with contrast (Case 5). Coronal view showing a cystic mass in the distal appendix. Pathological examination of the mass showed LAMN. LAMN, low-grade appendiceal mucinous neoplasm

Although LAMN can be generally asymptomatic and stable, it can also lead to a number of serious complications over time [1-3]. LAMN can rupture and disseminate mucin and neoplastic cells into the peritoneal cavity and result in PMP, an intraperitoneal accumulation of mucinous tumors and mucinous ascites that is associated with poor outcomes and high mortality risk [1-3]. Therefore, timely workup and management are essential to prevent progression to PMP and metastasis and improve overall outcome and recovery. Given it can be asymptomatic and have nonspecific findings when symptomatic, imaging is important for the initial detection of a mucinous appendix and surveillance for rupture. When intact, abdominal ultrasound can show a distended cystic appendix with porcelain wall calcification and a lamellated mucinous “onion-skin” appearance, though rupture will show an interruption in the appendiceal wall with leakage, while PMP will show thickening of the peritoneum or omentum, anechoic regions, echogenic foci, and septations [3]. CT can show an enlarged appendix with wall calcifications and thickening, and additionally demonstrate septations, calcified nodules, and liver margin scalloping in PMP (Figures 2-4) [1, 3]. Magnetic resonance imaging (MRI) can show a hyperintense distended appendix and bright mucin appearance on T2-weighted MRI, as well as nodularity on MRI with contrast [3]. A biopsy is not recommended due to perforation risk, though if the lesion has disseminated peritoneally, a biopsy of peritoneal nodules can be helpful for diagnosis [3]. Through these diagnostic measures, they can help to identify mucinous appendices, monitor for rupture, and allow for timely management to reduce the risk of dissemination and progression to PMP.

**Figure 5:**
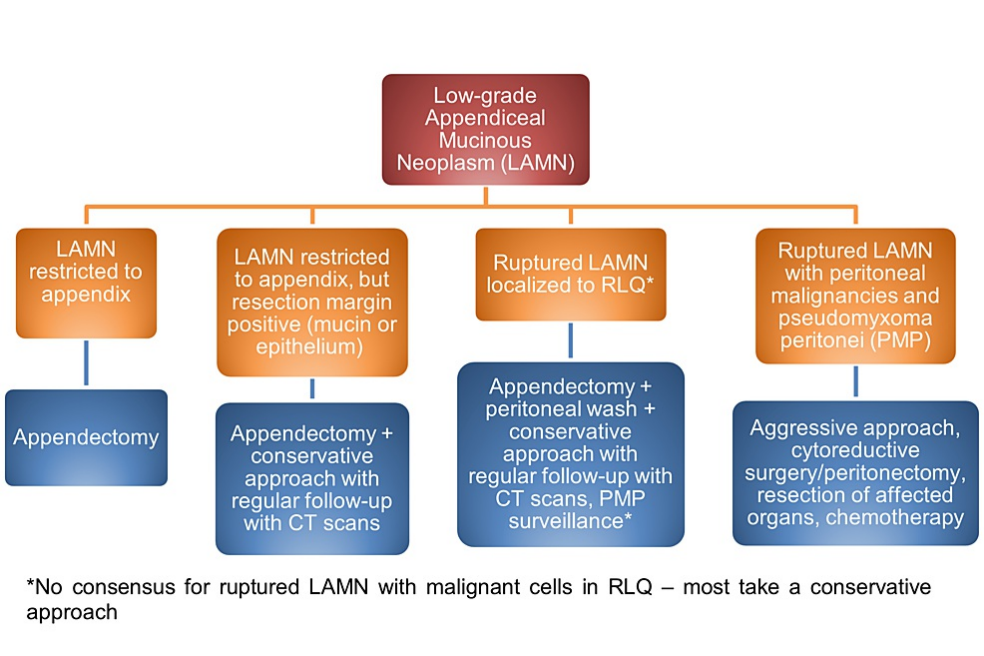
Algorithm for management of LAMN. LAMN, low-grade appendiceal mucinous neoplasm

To reduce risks of rupture and PMP progression and examine for the presence of LAMN pathologically, surgical removal of the appendix is crucial for further diagnosis and management [1-3]. Options for removal include open and laparoscopic resection. While open surgery is sometimes recommended to prevent LAMN rupture and PMP, there are documented cases of rupture and PMP in both open and laparoscopic resection with no comparative study available [5]. Including a cuff of the cecum without involving the ileocecal valve is preferred during appendectomy to ensure removal of the lesion [6]. Gross examination shows a cystically dilated mucinous appendix with a hyalinized, thin, fibrotic, or calcified wall and a smooth, corrugated, or granular lining (Figure 1) [1-2]. Because LAMN does not spread via the hematogenous or lymphatic route, an appendectomy is generally acceptable for the management of LAMN confined to the appendix on pathology, with conservative follow-up and surveillance if the appendectomy margins involve acellular mucin or neoplastic epithelium (Figure 5) [2, 7-8]. If acellular mucin is extruded on the appendiceal serosal surface without peritoneal dissemination, an appendectomy with close observation and evaluation for PMP via routine imaging appears to be sufficient [1-2]. Though recommendations on imaging frequency and duration may vary given there are no formal guidelines, we recommend annual CT imaging for five to ten years. Additionally, supplemental right hemicolectomy does not provide advantages over appendectomy in these patients and can involve the retroperitoneum, which may result in the seeding of the mucinous epithelium [2, 8]. If there is peritoneal dissemination, management may require appendectomy, irrigation, a biopsy of peritoneal nodules, peritonectomy, and chemotherapy [3].

**Figure 6:**
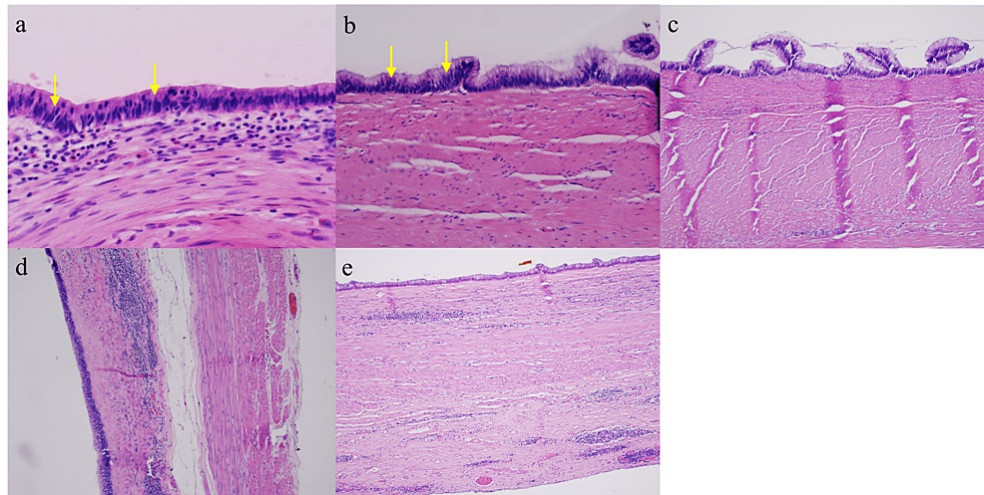
Pathology from Cases 1 to 5 (6a-e respectively). Neoplastic mucinous epithelium grows along the hyalinized stroma and plunges through muscularis propria, consistent with pushing invasion. Lamina propria is scant or absent. Neoplastic epithelium shows low-grade dysplasia with nuclear pseudostratification (arrows). Usual features of infiltrative type invasion, such as single-cell invasion, desmoplastic stromal reaction, and tumor budding are absent.

**Figure 7:**
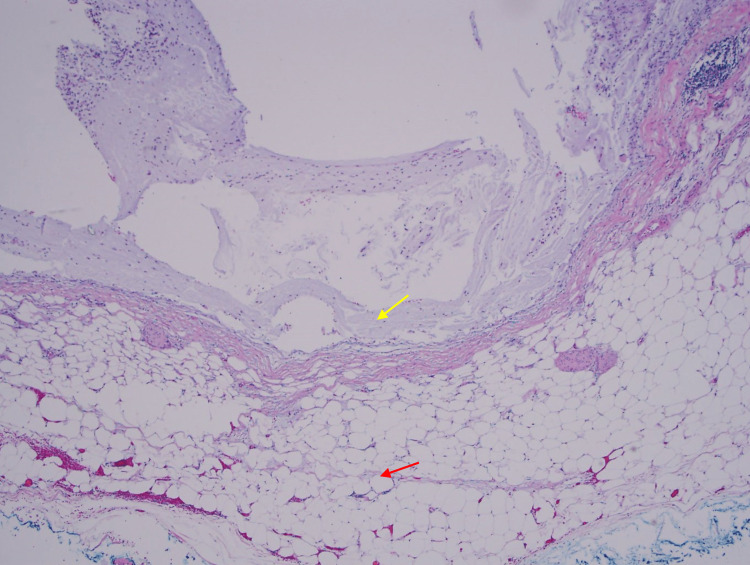
A microphotograph shows mucin sitting on the serosa (yellow arrow), which reached there by dissecting the wall (red arrow shows subserosal adipose tissue - the outer wall of the appendix).

To evaluate for the development of PMP and metastasis and assess whether LAMN is present, a pathological examination is needed for further analysis. While frozen sections are sometimes obtained for diagnosis and resection margin analysis, they may not be helpful for diagnosis given the complexity of LAMN pathology and the limited correlation of frozen sections with the final pathology [9]. Pathologically, LAMN is characterized by atypical glandular cells, neoplastic mucinous epithelium, back-to-back crypts, sparse lamina propria, and extended villi (Figure 6) [1-2]. It grows outward via “pushing” invasion, creating dissected or tongue-like epithelium and diverticula [2]. Mucin and neoplastic epithelium may dissect through the muscularis propria and even breach the appendiceal wall, seeding into the peritoneum and progressing to PMP (Figure 7) [2]. However, there are no signs of “infiltrative” invasion such as budding, single-cell invasion, or desmoplastic stromal reaction [2].

Staging is vital to examine for dissemination of mucin and neoplastic cells into the peritoneum and progression to PMP and metastasis. In pTis(LAMN), the LAMN is confined to the appendiceal wall, although acellular mucin or mucinous epithelial cells may still extend into the muscularis propria but not the mesoappendix or serosa [10]. In pT3, LAMN with acellular mucin or mucinous epithelium invades into the subserosa or mesoappendix but not the serosa [10]. In pT4, LAMN with acellular mucin or mucinous epithelium invades into the serosa of the appendix or mesoappendix (pT4a) or directly invades adjacent organs and structures (pT4b) [10]. In M1a, acellular mucin is disseminated intraperitoneally [10]. In M1b, metastasis occurs in the peritoneum [10]. In M1c, metastasis extends beyond the peritoneum [10]. If peritoneal metastasis or PMP is detected, the patient may require cytoreductive surgery (CRS), heated intraperitoneal chemotherapy (HIPEC), or early post-operative intraperitoneal chemotherapy (EPIC) [2,11]. CRS includes peritonectomy and clears away tumor cells to enhance the penetrability of HIPEC and EPIC [2,11]. After CRS and HIPEC, EPIC can be used to augment intraperitoneal therapy against tumor deposits [11].

Surveillance for recurrent disease and PMP after appendectomy is recommended [1]. While there is no established guideline regarding duration and frequency of surveillance, we recommend CT imaging annually for five to ten years and monitoring of tumor markers including CEA, CA 19-9, and CA-125. For LAMN restricted to the appendix with no extra-appendiceal mucin, appendectomy margins involving acellular mucin or neoplastic epithelium are not associated with recurrence of LAMN [7]. For pT4a LAMN, acellular mucin has a low risk of recurrence of 4%, while cellular mucin has a higher risk of recurrence of 23% [2,3]. Localized LAMN has a five-year survival rate of 95% [1]. Once LAMN progresses to PMP, prognosis will be poor, with a five-year survival rate of 25% [1]. Therefore, routine monitoring and timely management of LAMN are critical to reduce likelihood of LAMN recurrence and progression to PMP and metastasis.

## Conclusions

Low-grade appendiceal mucinous neoplasms are lesions of the appendix that can rupture and lead to PMP, an intraperitoneal dissemination of mucinous ascites and tumors with a high risk of mortality. For LAMN confined to the appendix, appendectomy alone is typically sufficient for management, with conservative follow-up and imaging if the margins involve acellular mucin or neoplastic epithelium. For LAMN with extruded acellular mucin localized to the appendiceal serosa, management is done with appendectomy and PMP surveillance. Peritoneal dissemination should be managed with appendectomy, a biopsy of peritoneal lesions, irrigation, and follow-up evaluation for CRS, HIPEC, and EPIC. With timely workup and management, this may help reduce the risk of complications, improve overall outcome, and increase the chance of survival and recovery.
